# The Chilean COVID-19 Genomics Network Biorepository: A Resource for Multi-Omics Studies of COVID-19 and Long COVID in a Latin American Population

**DOI:** 10.3390/genes15111352

**Published:** 2024-10-22

**Authors:** Iskra A. Signore, Gerardo Donoso, Pamela Bocchieri, Eduardo A. Tobar-Calfucoy, Cristian E. Yáñez, Laura Carvajal-Silva, Andrea X. Silva, Carola Otth, Claudio Cappelli, Héctor Valenzuela Jorquera, Daniela Zapata-Contreras, Yolanda Espinosa-Parrilla, Paula Zúñiga-Pacheco, Macarena Fuentes-Guajardo, Virginia A. Monardes-Ramírez, Pia Kochifas Velasquez, Christian A. Muñoz, Cristina Dorador, Jonathan García-Araya, Claudia P. Campillay-Véliz, Cesar Echeverria, Rodolfo Alejandro Santander, Leslie C. Cerpa, Matías F. Martínez, Luis Abel Quiñones, Eduardo Roberto Lamoza Galleguillos, Juan Saez Hidalgo, Estefanía Nova-Lamperti, Sergio Sanhueza, Annesi Giacaman, Gerardo Acosta-Jamett, Cristóbal Verdugo, Anita Plaza, Claudio Verdugo, Carolina Selman, Ricardo Alejandro Verdugo, Alicia Colombo

**Affiliations:** 1 Department of Anatomic Pathology, Faculty of Medicine, University of Chile, Santiago 8380453, Chile; isignore@uchile.cl (I.A.S.);; 2 Service of Anatomic Pathology, University of Chile Clinical Hospital (HCUCH), Santiago 8380453, Chile; 3 Human Genetics Program, Institute of Biomedical Science (ICBM), Faculty of Medicine, University of Chile, Santiago 8380453, Chile; 4 AUSTRAL-Omics, Vice Rector’s Office for Research, Development and Artistic Creation, Austral University of Chile, Valdivia 5090000, Chile; 5 Institute of Environmental and Evolutionary Sciences, Faculty of Sciences, Austral University of Chile, Valdivia 5090000, Chile; 6 Institute of Clinical Microbiology, Faculty of Medicine, Austral University of Chile, Valdivia 5090000, Chile; 7 School of Medicine, Magallanes University, Punta Arenas 6210005, Chile; 8 Evolutionary and Medical Genomics of Magallanes (GEMMa), Center for Education, Healthcare and Investigation (CADI-UMAG), Magallanes University, Punta Arenas 6210005, Chile; 9 Interuniversity Center for Healthy Aging, Punta Arena 6210005, Chile; 10 Department of Medical Technology, Faculty of Health Sciences, University of Tarapacá, Arica 1010197, Chile; 11 Clinical Laboratory of the Technical Area of Molecular Biology, Salvador Hospital, Santiago 7500922, Chile; 12 Department of Medical Technology, Faculty of Health Sciences, University of Antofagasta, Antofagasta 1240000, Chile; 13 Laboratory of Microbial Complexity and Functional Ecology, Antofagasta Institute & Biotechnology Department, University of Antofagasta, Antofagasta 1240000, Chile; 14 Laboratory of Molecular Virology, Faculty of Marine Sciences and Biological Resources, University of Antofagasta, Antofagasta 1240000, Chile; 15 Laboratory of Molecular Biology, Nanomedicine and Genomics, Faculty of Medicine, University of Atacama, Copiapó 1533601, Chile; 16 Emergency Public Assistance Hospital, Santiago 8330145, Chile; 17 Emergency Medical Assistance Service (SAMU), Punta Arenas 6200000, Chile; 18 Department of Basic and Clinical Oncology (DOBC), Faculty of Medicine, University of Chile, Santiago 8380453, Chile; 19 Latin American Network for Implementation and Validation of Clinical Pharmacogenomics Guidelines (RELIVAF-CYTED), 28015 Madrid, Spain; 20 Department of Pharmaceutical Sciences and Technology, School of Chemical and Pharmaceutical Sciences, University of Chile, Santiago 8380494, Chile; 21 Laboratory of Chemical Carcinogenesis and Pharmacogenetics (CQF), Department of Basic-Clinical Oncology (DOBC), Faculty of Medicine, University of Chile, Santiago 8380453, Chile; 22 Department of Computer Science, Faculty of Physical and Mathematical Sciences, University of Chile, Santiago 8370458, Chile; 23 Clinical Biochemistry and Immunology Department, Faculty of Pharmacy, University of Concepción, Concepción 4070383, Chile; 24 Center of Excellence in Translational Medicine, Faculty of Medicine, University of The Frontier, Temuco 4781176, Chile; 25 Institute of Veterinary Preventive Medicine, Faculty of Veterinary Sciences, Austral University of Chile, Valdivia 5090000, Chile; 26 Center for Surveillance and Evolution of Infectious Diseases, Austral University of Chile, Valdivia 5090000, Chile; 27 Institute of Animal Pathology, Faculty of Veterinary Sciences, Austral University of Chile, Valdivia 5090000, Chile; 28 Arturo López Pérez Foundation, Santiago 7500921, Chile; 29 Institute of Interdisciplinary Research, University of Talca, Talca 3460000, Chile; 30 School of Medicine, University of Talca, Talca 3460000, Chile

**Keywords:** biorepository, biobank networks, Latin American populations, COVID-19, underrepresented populations, genetic diversity

## Abstract

Although a lack of diversity in genetic studies is an acknowledged obstacle for personalized medicine and precision public health, Latin American populations remain particularly understudied despite their heterogeneity and mixed ancestry. This gap extends to COVID-19 despite its variability in susceptibility and clinical course, where ethnic background appears to influence disease severity, with non-Europeans facing higher hospitalization rates. In addition, access to high-quality samples and data is a critical issue for personalized and precision medicine, and it has become clear that the solution lies in biobanks. The creation of the Chilean COVID-19 Biorepository reported here addresses these gaps, representing the first nationwide multicentric Chilean initiative. It operates under rigorous biobanking standards and serves as one of South America’s largest COVID cohorts. A centralized harmonization strategy was chosen and included unified standard operating procedures, a sampling coding system, and biobanking staff training. Adults with confirmed SARS-CoV-2 infection provided broad informed consent. Samples were collected to preserve blood, plasma, buffy coat, and DNA. Quality controls included adherence to the standard preanalytical code, incident reporting, and DNA concentration and absorbance ratio 260/280 assessments. Detailed sociodemographic, health, medication, and preexisting condition data were gathered. In five months, 2262 participants were enrolled, pseudonymized, and sorted by disease severity. The average Amerindian ancestry considering all participant was 44.0% [SD 15.5%], and this value increased to 61.2% [SD 19.5%] among those who self-identified as Native South Americans. Notably, 279 participants self-identified with one of 12 ethnic groups. High compliance (>90%) in all assessed quality controls was achieved. Looking ahead, our team founded the COVID-19 Genomics Network (C19-GenoNet) focused on identifying genetic factors influencing SARS-CoV-2 outcomes. In conclusion, this bottom-up collaborative effort aims to promote the integration of Latin American populations into global genetic research and welcomes collaborations supporting this endeavor. Interested parties are invited to explore collaboration opportunities through our catalog, accessible online.

## 1. Introduction

The problem of low ethnic diversity in research has been highlighted as a major obstacle to the advancement of personalized medicine and precision public health, leading to inaccurate risk evaluations, inadequate public policies, and health inequalities [1,2]. Although Asian representation in biorepositories and Genome-wide association studies (GWASs) has notably increased in the last decade, African and Latin American populations are still underrepresented [3,4]. This is also the case for COVID-19, despite its enormous variability in clinical course and severity depending on the patient. Since its first description, a broad spectrum of clinical manifestations [5,6] and several risk factors affecting disease severity have been reported [7], and the importance of a personalized approach has been recognized [8,9,10]. GWASs have shown an association between host genetic architecture and susceptibility to COVID-19 [11]. Nevertheless, the Centers for Disease Control and Prevention reported that in the United States, the COVID-19 hospitalization rate is higher for non-Hispanic Amerindians or Alaska Natives, non-Hispanic black people, and Latinos when compared with non-Hispanic white people [12]. Also, Shelton and colleagues (2021) found that the hospitalization rate for African Americans was nearly double what would be expected based on the proportion of positive COVID-19 tests among the customers of a direct-to-consumer testing company. Remarkably, non-European ancestry is a risk factor for hospitalization even after adjusting for sociodemographic and preexisting health conditions [13]. This difference cannot be explained by the two primary known genetic associations (ABO and 3p21.31 loci), suggesting the presence of other risk loci in the genome with ancestry-related allele frequency variations. The COVID-19 Host Genetics Initiative published two updated meta-analyses from July 2021 to date, reporting a total of 51 genome-wide loci significantly associated with SARS-CoV-2 infection or severe COVID-19. The inclusion of European, admixed American, African, Middle Eastern, South Asian, and East Asian individuals in these reports is particularly noteworthy since GWASs for COVID-19 have primarily been performed on populations of European ancestry [14,15,16]. This initiative is playing a crucial role in expanding collaboration globally, but according to information on its website [17], of the 119 registered partner studies, only three are from South America (Brazil, Paraguay, and Chile) and two are from Mexico. Similarly, 11 studies are based in Asia, and Africa and Oceania each provide one study. Apart from the five Canadian partner studies, all the others are performed in Europe and the United States. Despite their limited numbers, studies in underrepresented populations have proven useful in identifying genetic traits significant to these populations that are not detected using trans-ancestry analyses. The genes DOCK2 in East Asians, IFNAR1 in Polynesians, and IFNAR2 in the Inuit population are examples of such findings [18,19,20]. To the best of our knowledge, only four genetic studies on COVID-19 in Latin American populations (Brazil and Colombia) have been published to date [21,22,23,24]. This scenario highlights the importance of generating and providing resources for collaborative efforts in under-studied populations. 

In addition to the issue of population representativeness, other critical challenges for precision and personalized medicine are the reproducibility of results and the availability of a sufficient quantity of high-quality samples and data to perform reliable analyses. The reproducibility problem is considered common across all scientific disciplines [21,22,23], but addressing it in biomedicine requires, among other things, using comparable samples. This means obtaining samples and data with harmonized and robust methods, minimizing pre-analytical variables through standardized processes, the use of alternative methods, and exhaustive documentation [24,25]. The logistical and expertise burdens required to create and organize a robust collection of biological samples annotated with clinical and demographic data are usually beyond the reach of individual researchers and their groups, and this is a significant reason for the inconsistent quality of collections [26]. Biobanks are pivotal in addressing this challenge [27,28,29] by managing biological samples and their associated data with high-quality standards and monitoring. They ensure these samples and data are rapidly available, traceable, and compliant with ethical and legal requirements. Thus, biorepositories built by biobanks have a particularly high value as resources for scientific collaboration and public interest.

During the COVID-19 pandemic, biobank networks rapidly supported global initiatives by providing the scientific community with samples and data collection and conservation, together with providing standards and guidelines [30,31]. For example, The United Kingdom Biobank and the pan-EU Biobanking and Biomolecular Resources Research Infrastructure (BBMRI-ERIC) generated large COVID-19 collections to study seroprevalence, its relationship with ethnicity, and comorbidities [13,30,31,32,33]. However, in Latin America, biobanks are still underdeveloped and need to be strengthened. Their synergy with researchers, policymakers, and biotech companies is essential for building clinical strategies and public health policies that match the real characteristics of the target population. This paper reports the articulation of a network across Chile and the creation of the Chilean COVID-19 Biorepository, the first nationwide multicenter repository built under biobanking standards to ensure the high quality, reproducibility, and interoperability of samples and data as a resource for collaborative multi-omics research.

## 2. Materials and Methods

### 2.1. Network Establishment and Harmonization

**Figure 1:**
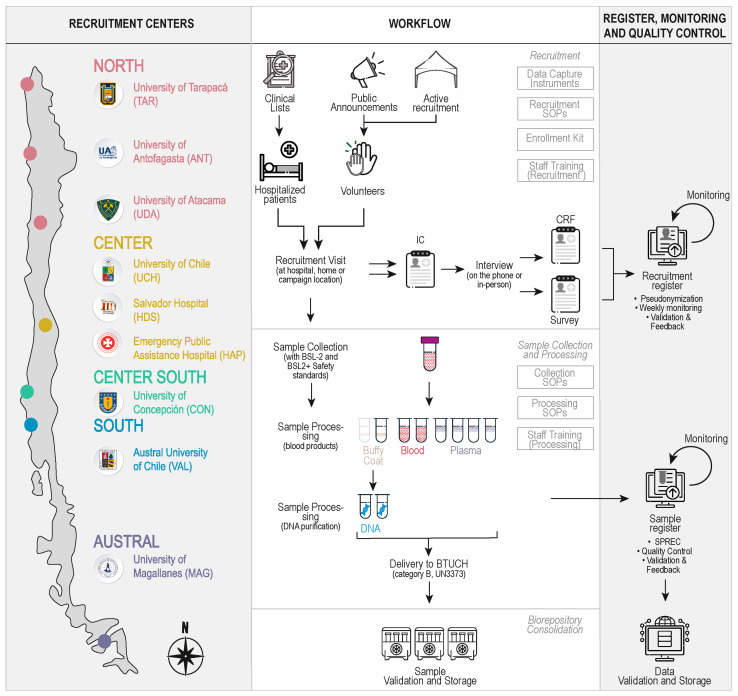
The Chilean COVID-19 Biorepository design and workflow. (**Left panel**). Countrywide distribution of recruitment centers. Macrozones and the nine centers are located throughout the country, from the northern border to the southern region of the country. (**Center Panel**). Workflow of the biorepository implementation showing recruitment (**top**), sample collection and processing (**middle**), and consolidation (**bottom**). Processes performed in each phase are shown in boxes at the right corner of each subpanel. Volunteers joined the study through both active recruitment organized by centers and public announcement campaigns on social networks and the web. The IC, CRF, and survey were completed in person (at the centers or at the participant’s home), by telephone, or from the clinical records according to the requirements of each case. Processing of samples consisted of two phases. Blood samples were processed to obtain plasma (single spun) and buffy coat, and then DNA from the latter. A complete set of samples included two tubes of blood (1 mL each), four of plasma (250 µL each), one of buffy coat with 500 µL of RNA-later (Thermo Fisher Scientific, Waltham, MA, USA), and two of DNA per donor (50–100 µL each). In 55 cases, poor venous access was noted in patients, so the sample was collected by buccal swabbing and entirely used to purify DNA, representing the only stored specimen in these cases. (**Right Panel**). An online register system was implemented for monitoring and quality control. Data from the CRF and survey were collected in the recruitment register, whereas sample data were fed into the sample register. Registers were monitored and validated weekly to achieve the goals of the Chilean COVID-19 Biorepository (see the text for details).

The Chilean COVID-19 Biorepository was established when, after the National Research and Development Agency of the Ministry of Science (ANID) announced a competition in May 2020 for rapid funding allocation for COVID-19 research projects, four principal investigators involved in this project were awarded funds for studies utilizing human samples. None of the projects were aimed at the creation of biorepositories, recruitment centers, or biobanking operations. However, it became readily evident that to ensure the acquisition of high-quality samples and data for ongoing research, as well as to bolster the national response to the pandemic optimizing resources, fragmented efforts in building individual collections were inefficient and risky given the difficulty and required expertise. The University of Chile’s Biobank of Tissue and Fluids (BTUCH) was consulted as one of the few established and experienced biobanks in the country. In this way, BTUCH assumed the articulating role that led a group of individual researchers from various institutions nationwide to start this bottom-up collaborative effort. We decided to pool resources, operate jointly, and fund the collection of samples and data according to biobank standards, thereby organizing a network of recruitment centers throughout the country and the creation of this national biorepository of COVID-19. Thus, the Chilean COVID-19 Biorepository was finally established through a network of nine nodes across seven of the country’s 16 regions, encompassing all macrozones (Figure 1, left panel).

Although the network is federated, a centralized intensive strategy was chosen for operational harmonization. Before recruitment began, an intensive harmonization phase for the operational installation of the network was carried out for one month under the direction of the BTUCH. This process included the creation and implementation of unified (i) standard operating procedures (SOPs) and registers, (ii) sample coding system, and (iii) staff training across all centers.

Unified quality control parameters were established and implemented according to biobank standards, operationally defined through common SOPs and registers. The Standard Preanalytical Code (SPREC) [34] was adopted to manage and document pre-analytical variables, and a minimum DNA concentration standard of 30 ng/µL was set, as it is suitable for most technical needs, including sequencing. Common procedures and registers for incidents and standard sample quality parameters were also established (see Sample Collection and Processing section for details). Common instruments for the collection of data for the participants’ characterization were also created and included a survey and case report form (CRF). These instruments were constructed based on existing publications, documents already used by our researchers, and by national healthcare centers. Our symptom list was compiled from the literature, the COVID-19 Participant Experience-COPE [35], and a preliminary LMIC LPS COVID-19 Questionnaire from the Wellcome and the International Hundred Cohort Consortium-IHCC group provided by personal communication with Dr. Teri Manolio. For clinical data nomenclature, LOINC and SNOMED CT standards were used where applicable [36,37]. Another harmonization choice was made to prevent problems due to ambiguous, incorrect, or inconsistent associations among codes, participants, their data, and samples. The adopted strategy was designed to fully centralize the process by preparing individual recruitment kits for each participant. These kits included all necessary documents (informed consent, case report form, epidemiological survey, and quality control records) and containers, each bearing pre-affixed codes. BTUCH centrally prepared, validated, and dispatched all enrollment kits to each center. Documentation was also centrally indexed in a shared online document management register.

The preparatory harmonization took a month and was followed by a pilot recruitment phase performed at Salvador Hospital in September 2020 for reality-based adjustments. Weekly general network meetings were held throughout the construction of the biorepository to review quality parameters and recruitment dynamics, enabling the timely detection and resolution of difficulties or inconsistencies in each center’s performance.

### 2.2. Ethics Approvals and Informed Consent

The Chilean COVID-19 Biorepository received approval from the University of Chile’s Faculty of Medicine Ethics Committee and additional ratification from local Ethics Committees at each participating site (Ethics Committees of the University of Magallanes, Central Metropolitan Health Service, University of Tarapacá, and Scientific Ethical Committee at Valdivia Health Service). Following international ethical recommendations [33], the network decided to use a broad informed consent (IC) adapted for biobanks. This type of IC allows the storage and use of samples and data for this and future studies on COVID-19 and other types of biomedical research, as well as recontacting the participant for follow-up assessments and obtaining further samples and data. The management of donor recruitment, samples, and data was carried out in compliance with national laws [38,39], international ethical and legal regulations [40,41,42], and good biobank practices [43].

### 2.3. Study Design and Recruitment

The study included individuals over 18 who provided written informed consent to participate and be recontacted. Enrollment methods included hospitalized patient invitations and volunteer recruitment (Figure 1, top center panel). Only adult hospital patients with positive nasopharyngeal qRT-PCR tests were included, given laboratory confirmation accessible via electronic records. Patients admitted for other reasons and circumstantially found to be COVID-19 positive were excluded. For non-hospitalized patients, confirmed infection of SARS-CoV-2, either by laboratory testing (SARS-CoV-2 qRT-PCR test, SARS-CoV-2 rapid antigen test, or antibody tests for COVID-19) or clinical diagnosis, was required. Patients self-reporting suspected disease were not included in the study. Samples were collected and processed in compliance with biosafety guidelines for levels 2+ and 2 (BSL-2 and BSL2+) [44]. The delivery of samples used a triple packaging system, according to the international guidelines for biological substance category B, UN3373 [45].

**Figure 2:**
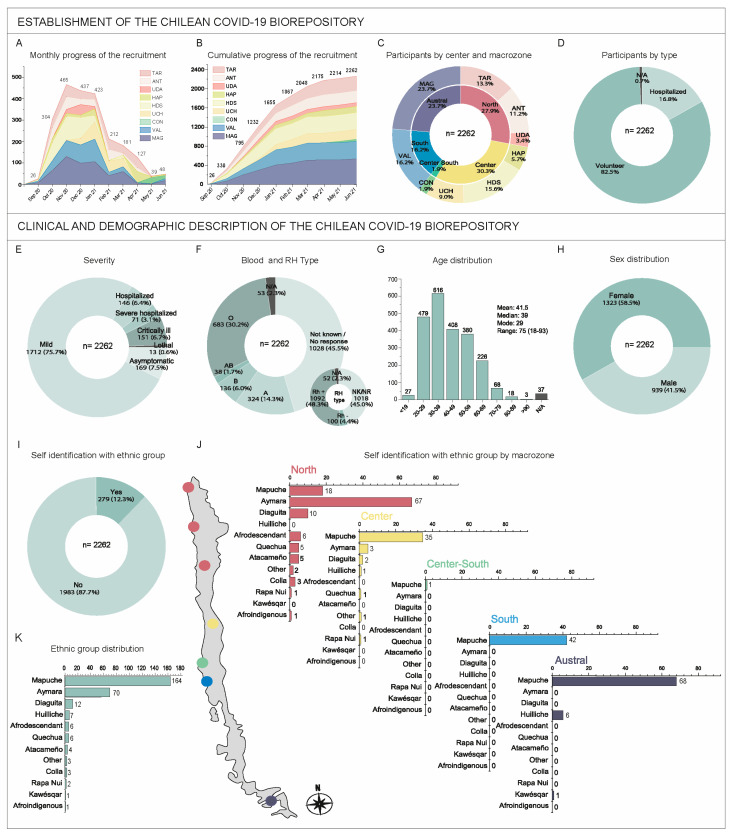
Description of the dynamics of recruitment and donors’ information of the Chilean COVID-19 Biorepository. (**Top panel**) Establishment of the Chilean COVID-19 Biorepository (**A**,**B**) Recruitment progress. Cases registered in September 2020 correspond to the pilot phase (see text), while those who appear during June 2021 correspond to donors whose date of capture was corrected a posteriori or those who presented errors, inconsistencies, or incomplete data. (**C**) Concentric graph showing in the inner ring the distribution of participants captured by macrozones in correspondence with related centers shown in the external ring. (**D**) Proportion of participants entered through volunteer campaigns or hospitalization. The cases indicated as N/A (*n* = 15) correspond to those data that presented errors or inconsistencies or were absent. (**Bottom panel**) Clinical and demographic description of the biorepository. (**E**) Proportion of patients according to severity. (**F**) Self-reported blood and RH type. NK: not known, NR: no response. (**G**) Histogram depicting the age structure of the biorepository cohort. The descriptive statistical parameters mean, median, mode, and range are shown, with the youngest patient being 18 years old and the oldest being 93 years old. The 44 cases labeled N/A correspond to cases with inconsistent, erroneous, or missing data, as noted in the previous panel. (**H**) Proportion of female and male participants in the biorepository showing that most of them were women. (**I**) In total, 386 participants self-identified as belonging to an ethnic group. Cases labeled N/A correspond to those with inconsistent, erroneous, or missing data. The frequencies of each group at the whole biorepository level are shown in (**K**), sorted from highest to lowest. The order obtained is maintained in (**J**) to show the distribution of these ethnicity groups within each macrozone.

Each center maintained an online registry for weekly recruitment optimization. Trained personnel gathered IC and subsequent CRFs or surveys (Figure 1, top center panel) to capture relevant data (see Section 2.4 and Section 3.2, Figure 2, and Table 1 and Table 2). After local review, the pseudonymized and digitized data were validated centrally and logged into the recruitment register, the weekly monitoring of which allowed for continual improvement (Figure 1, top right panel) and tailored strategies for each center’s challenges.

### 2.4. Data Collection and Management

The survey and CRF were built using comparable modularization. The modules for data were personal and sociodemographic characterization, lifestyle habits, clinical data related to COVID-19 symptoms and their onset date, treatments received, and pre-existing conditions (Section 3.2, Figure 2, Table 1 and Table 2). Staff in charge of interviewing participants were trained to harmonize communication criteria during interactions, ensure the ethics of the process, and minimize errors and missing data. In some centers, data capture was done directly on portable digital devices, while in others lacking such technology, paper documents were used with subsequent digitization. This procedure required adding a verification and validation step for the digitized data. During recruitment, data were locally reviewed and logged into the recruitment register or sample register as appropriate and centrally monitored remotely by the BTUCH. Monitoring results were analyzed during the weekly meetings for feedback and adjustments.

The software used to construct the final database containing participants’ data was the RedCAP platform (version 10.8.3). This biomedical research platform that is commonly used in biobanking has a nonprofit license, is highly configurable, is user-friendly for those without advanced computing skills, and is also free [46,47]. The staff of the centers was trained to use it according to their need. The RedCAP’s monitoring module was used for data quality control by the BTUCH to check for plausibility, conformance, and completeness [48]. The complete list of available data is available on request by contacting the authors. The catalog of samples was created using the NorayBanks Catalogue^®^ software (version 1.30.2310.1804), and it is accessible at https://redcovid.uchile.cl/.

### 2.5. DNA Genotyping and Estimation of Ancestry

Genome-wide genotypes were generated by hybridization to Global Survey Arrays v3 (Illumina, Inc, San Diego, CA, USA). Individuals and SNPs with over 5% missing data, *p*-values < 10^−6^ for Hardy–Weinberg disequilibrium, and MAF < 0.01 were excluded. Genotypes were merged with a reference data set of 40 European (CEU), 40 African (AFR), and 40 East Asian (EAS) individuals from the 1000 Genomes Project [49], 40 individuals of Aymara ancestry (personal communication, Andrés Moreno-Estrada), and 40 individuals of Mapuche ancestry [50,51]. Global ancestry was estimated using ADMIXTURE v1.3.0 with supervised clustering [52]. The best number of estimated subpopulations (*k*-value) was chosen to minimize cross-validation error.

### 2.6. Participants Recontact for Long COVID Follow-Up

For the long COVID follow-up, we attempted to recontact 2249 participants for a survey with standardized practices in place. After three phone calls and additional follow-up, 1688 (75.06%) were reached. Among them, 111 (4.94%) declined, 191 (11.32%) partially completed, and 1386 (82.11%) fully completed the survey. The high response rate highlights effective recruitment and recontact efforts.

### 2.7. Patient and Public Involvement

Participants were not involved in the design stage design but provided feedback during recruitment. We arranged home visits and matched recruitments with medical appointments to ease participation. We incorporated an online survey option sent via email or chat. To foster trust, we listened to data return preferences, resulting in personalized ancestry analysis reports for participants.

## 3. Results

### 3.1. Establishment of the Chilean COVID-19 Biorepository

In 8 months, 2262 participants were enrolled. Figure 2A,B shows the recruitment progress, revealing that dynamics varied. The most intense recruitment period was between October 2020 and February 2021, with a peak of 465 participants in November 2021. Despite the high centralization of the country in terms of population, accessibility, and general resources, macrozone contributions were impressively even (Figure 2C), barring Center South (contribution of 1.9%). Regarding admission routes, 1866 donors were volunteers, while 381 were hospitalized patients (Figure 2D). The remaining 15 cases have missing or inconsistent information on this point (0.7%, indicated as N/A in Figure 2D).

### 3.2. Description of the Chilean COVID-19 Biorepository

Participants were categorized by disease severity into six categories: asymptomatic (169); mild (1712); hospitalized (146, not requiring oxygen support); severe hospitalized (71, requiring oxygen support); critically ill (151, requiring respiratory support with mechanical ventilation such as continuous (CPAP) or bilevel (BiPAP) positive airway pressure or Optiflow/very high-flow positive end-expiratory pressure oxygen (PEEP)); and lethal (13) (Figure 2E).

Using the survey and CRF, we collected data on participants’ sociodemographic characteristics (sex, age, weight, height, level of schooling, private/public healthcare, self-identification with ethnic groups, and ancestry), habits (tobacco, alcohol, and drug consumption), clinical information (blood group, body mass index, use of medications, chronic and preexisting pathologies, COVID-19 symptoms and treatment, and others). Figure 2 shows some of these data. Self-reported blood and Rh type showed that a large proportion of individuals did not know their ABO and Rh blood group. Nevertheless, the most common types in the biorepository are O and Rh+ (Figure 2F). The age distribution and its descriptive statistics are shown in Figure 2G; most participants were between 30 and 39 years old. Females account for 58.5% of the biorepository (Figure 2H).

A noteworthy feature is the presence of 279 self-identifying members of an ethnic group (12.3% of the repository) (Figure 2I). Members of eight of the ten officially recognized native peoples in Chile were present, along with Huilliche, Afro-descendants, and Afro-Indigenous (Figure 2K). Figure 2J shows the distribution of ethnic groups by macrozones, and some geographic correlations between the two can be appreciated.

### 3.3. Description of Sample Collection

**Figure 3:**
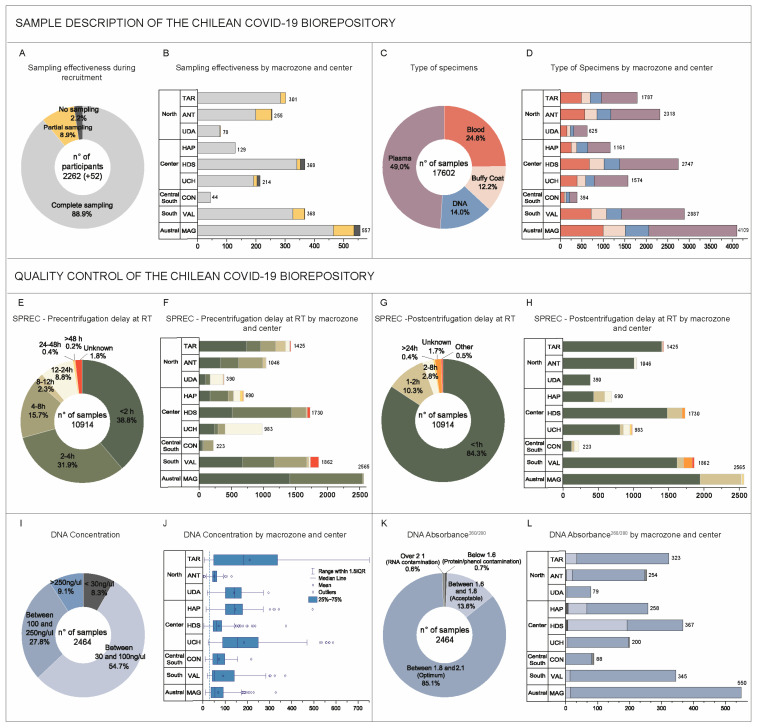
Description of the type and quality of the biological samples of the Chilean COVID-19 Biorepository. (**Top panel**) Sample description. (**A**,**B**) Performance of the centers concerning the collection of donor samples. Only 52 participants were left out of the biorepository due to sample collection failure (in black). For the remaining 2262 cases, a final set of 8 or 9 sample tubes was considered a complete sampling since it includes all necessary specimen types (see Figure 1). Donors with a final set of between 1 and 7 specimens were considered partial sampling. (**C**,**D**) Distribution of specimens by type in the whole repository and by macrozones and centers of the whole repository. (**Bottom panel**) Quality control. (**E**–**H**) Standard preanalytical code (SPREC) for pre-centrifugation (**E**,**F**) and post-centrifugation (**G**,**H**) delay at room temperature. Compliance with the required standard is depicted in all shades of green, while values below the acceptable standard are indicated in yellow, orange, and red and correspond to values larger than 24 h for both pre- and post-centrifugation as well as incompletely recorded cases (labeled as unknown) and those with other notes that do not correspond to any other SPREC code (labeled as other). (**I**–**L**) DNA quality in terms of concentration (**I**,**J**) and purity (**K**,**L**). Samples that meet the standard established for the biorepository are shown in shades of blue, while non-compliant samples are noted in gray. In (**J**), the dotted line corresponds to the minimum concentration standard of 30 ng/µL.

The biorepository is physically located at BTUCH within the Clinical Hospital of the University of Chile in Santiago (coordinates −33.4199, −70.6531). For 2057 out of 2262 participants (88.9%), we collected a complete set of biospecimens, while at least one sample was collected from 205 donors (8.9%). No sample was obtained in only 52 cases (2.2%) (Figure 3A). The average effectiveness of centers in obtaining the entire set of biospecimens per patient was 91.7% (minimum value 78.0%), while the average of obtaining no sample per patient was 1.6% (maximum value 4.7%). The high capture effectiveness shows that our multicenter design achieved good ongoing national coordination (Figure 3B,D).

The total number of biospecimens is 17602, distributed as follows (cryotubes/different samples: blood (4357/2198), buffy coat (2150/2150), DNA (2464/2186), and plasma (8631/2159) (Figure 3C). Incidents were reported only in 82 cases (3.6%) and included hemolyzed (*n* = 36), lipemic (*n* = 24), icteric (*n* = 10), and clotted *(n* = 2) samples, plus 10 tubes in which the minimum collection was not achieved.

We performed QC assessments according to SPREC, confirming the proper handling of the samples. However, our effectiveness in recording preanalytical information was lower. Out of 17,602 total samples, we have 10914 quality records, of which only 266 (2.4%) and 282 (2.6%) did not meet pre- and post-centrifugation delay requirements, respectively (Figure 3E–G sum of yellow, orange, and red). For pre-centrifugation delay, only three centers performed under the 95.0% compliance benchmark (Figure 3F; 92.8% HAP, 93.0% VAL, and 94.9% UDA), while only one center did not meet the benchmark for post-centrifugation delay (Figure 3H; 91.7% VAL).

Our minimum standard for the DNA concentration was 30 ng/µL, suitable for most subsequent technical needs, including sequencing. Considering the whole repository, 2259 samples out of 2464 fulfilled this requirement, reaching 91.7% compliance (Figure 3I, all shades of blue) with an average DNA concentration of 109.1ng/µL. Remarkably, 37.0% of the DNA samples (911 biospecimens) had a concentration above 100 ng/µL (Figure 3E). Only 3 out of 9 centers had an average compliance rate below 90% (60.5% MAG, 77.6% TAR, and 88.8% VAL) for the 30 ng/mL standard, but all centers achieved a mean concentration well above this limit, as shown in Figure 3J. The analysis of the Absorbance 260/280 of DNA samples revealed that only 32 samples (1.3%) were contaminated with protein, phenol, RNA, or other contaminants (Figure 3K, in shadows of grey). Most DNA samples (2098 specimens) showed optimal quality with absorbance between 1.8 and 2.1, while 334 had acceptable quality with absorbance between 1.6 and 1.8 (Figure 3K, all shades of blue). The performances of the different centers were once more even, with 93.2% of uncontaminated samples being the lowest level of compliance regarding DNA absorbance values (Figure 3L).

### 3.4. Description of Ethnic Composition and Ancestry

When writing this paper, validated and quality-controlled ancestry analyses were completed for 2099 participants. The remaining 163 (7.2%) failed sequencing or had inadequate DNA and were excluded from the run. These participants have been recontacted, and new samples were taken from consenting individuals and are undergoing analysis. Thus, the ancestry of the whole cohort was determined using genome-wide genotyping data from 92.8% of the samples, resulting in an average distribution of 2.0% African [SD 3.6%], 1.6% Asian [SD 4.2%], 52.4% European [SD 15.2%], and 44.0% Amerindian [SD 15.5%] participants. The latter ancestry can be decomposed into 13.4% [SD 17.2%] representing a northern component more closely related to Aymara and Quechua ethnicities and 30.6% [SD 15.0%] representing of a southern component more closely related to Mapuche ethnicity. The Amerindian component was 61.2% [SD 19.5%] among individuals who self-identified with any Native American ethnic group. Details on ancestry by self-declared ethnicity can be found in Table 1.

### 3.5. An Example of Collected Clinical Data: Description of Symptoms and Comorbidities

Although it is true that all the data could eventually be of interest, in order for all the centers to be able to capture these data, clinical data to be recorded were harmonized as well. However, without intending to establish any analysis of results and only as an example, we present the frequencies of symptoms and comorbidities recorded in this section to give an idea of the sub-cohorts that could be of interest to the scientific community. Other data recorded included information on the use of tobacco and other psychotropic drugs and type of health care, among others. The complete list of variables is available on request from the network catalog or by contacting the authors. Participants reported 34 previously described symptoms. Symptoms ranged in frequency from 3.0% to 68.1% for skin discoloration and fatigue, respectively. Table 2 shows the five most common symptoms by severity level. Coherently, respiratory symptoms like dyspnea (both at exertion and rest) were increased in more severe disease forms, leading to hospitalization. A complete list of symptoms and detailed frequencies are available in Supplementary Table S1. Also, 26 comorbidities were reported, and obesity, hypertension, diabetes, high cholesterol, and other chronic diseases were found in all conditions. The five most common comorbidities by severity are also shown in Table 2, and the full list is available in Supplementary Table S2.

## 4. Discussion

Here, we report one of Latin America’s largest biobank cohorts for COVID-19, alongside BRACOVID (5233 participants) [53]. Noting Brazil’s eleven-fold greater population, the Chilean COVID-19 Biorepository is a remarkable effort. The cohort design targets genetic variation associated with COVID-19 severity in a nationwide, admixed ancestry cohort closely matching Chile’s general genetic profile [54,55]. Notably, the COVID-19 Host Genetics Initiative and 23andMe GWAS studies showed 71–78% [14] to 80.3% [56] European ancestry, paralleled by the Million Veteran Program reporting 70% [57]. This underlines the importance of Latin American biorepositories like ours, showing a European composition of 22.9–54.7%, depending on self-identified ethnicity (Table 2).

Although challenging, the project’s nationwide multicenter structure aimed to ensure consistent sample and data quality, counteracting biases from recruiting participants in very large versus small urban centers and differences in site experience, especially since several were working with biobank quality management standards for the first time. The fundamental strategic pillars we adopted for this included centralizing the harmonization process, ensuring its thoroughness and completeness in terms of procedures and documentation management, standardizing processes, unified staff training before recruitment began, and a continuous monitoring strategy for quality management throughout the project. For example, by monitoring the quality of the samples obtained, a change in the DNA extraction process was implemented, favoring purification from the buffy coat over purification from blood. Also, the need for re-training personnel in charge of DNA purification in some centers was detected early through quality parameter reviews of the samples, promptly improving their competence for this specific task. Our experience shows that while standardization of biobanking processes is paramount for good results, it alone cannot replace or offset the necessity for expert personnel whose training and oversight must be consistent and continuous across the entire network. Our design led to efficient blood sample collection and high-quality blood products (Figure 3A), including satisfactory DNA concentrations (Figure 3I), as confirmed with SPREC QCs. Nevertheless, future efforts should focus on enhancing the capture of this information. This might include raising awareness among personnel and researchers about the significant effects of preanalytical variables on the samples and, consequently, the importance of their rigorous handling and documentation.

Weekly monitoring also optimized data quality and completeness by allowing participants to be recontacted for CRF or survey issues. This strategy allowed us to obtain a very low number of inconsistencies, absences, or errors in data collection (indicated as N/A in Figure 2 and Figure 3) that affected a maximum of 53 cases, corresponding to less than 2.5%. Our results show that our strategies worked in achieving the aimed composition and quality of the Chilean COVID-19 Biorepository.

Three years after the COVID-19 pandemic, the disease’s severity prediction is still complex. Confirmed risk factors are age and sex, with older males reporting higher severity [58]. Preexisting conditions like hypertension and diabetes tend to elevate the severity, yet the comorbidities’ exact correlation with prognosis is unknown. Although the effect of the blood group on clinical outcomes remains unclear, its influence on infection susceptibility is probable (reviewed in [59]). Figure 2 and Figure 3 illustrate that our biorepository could provide relevant biospecimens and data to study these factors, among other epidemiological or lifestyle variables that we registered.

The use and acceptance of broad informed consent are established but only rather recently in the country. Although an authorized committee’s ethical approval in our legislation permits national studies [38], new revisions by the ethics committees at each recruitment center were required, delaying the project and reducing recruitable participants within budget. Thus, balancing local particularities with national policy progression is crucial for safeguarding participants’ rights and welfare while allowing faster study execution, particularly when beneficial for public strategy direction during pandemics [33,60].

Motivating participation in scientific studies is an ever-evolving challenge closely related to a society’s valuation of science. In Chile, science is not a principal cultural or social value, fostering an environment of uncertainty and low levels of cooperation. We believe that responsible research and innovation should be integrated into scientific thinking as a social necessity for development. Greater societal appreciation of science should facilitate access to the sources of information that generate knowledge, which in this case are the participants themselves. Biobanks play a crucial positive role in this process, but Chile has no national policy for their formation and operation, leading to a lack of direct or competitive funding. Thus, it is difficult to maintain even the few existing biobanks, and the lack of them in areas other than the capital limits the storage infrastructure and makes it easier for valuable biological material to be discarded.

### 4.1. Limitations of This Work

Although the collection comprised 2262 cases, particular ancestry groups like Aymara or Mapuche constitute only a small sample subset. Similarly, very severe COVID-19 cases are less represented. Another limitation of the work is that it is based on a self-reported survey, which may represent a bias when analyzing specific clinical phenotypes. Also, we recruited participants within 1 to 10 months after SARS-CoV-2 infection. This temporal heterogeneity may hamper the comparison of some parameters among cases due to transcriptional dynamics after SARS-CoV-2 infection [61] or the ability to perform specific analyses, such as some kinetic analyses in plasma samples taken too late after infection. Similarly, since most of the recruited cases suffered from mild COVID-19, very severe COVID-19 may be underrepresented in our cohort.

### 4.2. Future Perspectives

Thanks to the publication of this work, we expect that this repository will attract global network collaborations for comparative studies on the effects of COVID-19 across diverse populations, including exploring potential genetic advantages or disadvantages in the context of SARS-CoV-2 infection. For example, in collaboration with the COVID-19 Host Genetics Initiative, the Chilean COVID-19 Biorepository has contributed to the publication of the second updated genome-wide association study, further enhancing our knowledge of the role of host genetics in the susceptibility and severity to SARS-CoV-2 [16].

Researchers involved in establishing this biorepository are currently associated with a collaborative research initiative known as the COVID-19 Genomics Network (C19-GenoNet), aiming to accelerate the identification of genetic factors in both hosts and pathogens that influence the short- and long-term outcomes of SARS-CoV-2 infection. The broad informed consent we used enables longitudinal cohort follow-up, thereby allowing for investigating the long-term consequences of SARS-CoV-2 infection, particularly concerning long COVID. Our recontact and recruitment procedures have so far yielded a high response rate (82.11%), demonstrating strong participant engagement. This long COVID cohort has been leveraged by collaboration with the COVID-19 Host Genetics Initiative for the forthcoming publication of a genome-wide association study on long COVID. We are also collaborating with the Spanish Coalition to Unlock Research on Host Genetics on COVID-19 (Scourge), providing genomic, demographic, health, and lifestyle data. As we move forward, this initiative aims to integrate Latin American populations into global genetic research and seeks collaborations that will mutually enhance, strengthen, and expand the research capacities of all participants. We invite interested parties to explore partnership opportunities through our catalog, which is accessible at https://redcovid.uchile.cl/ or by contacting the authors.

## 5. Conclusions

This biorepository has potential national and international public health impact, addressing the previously highlighted issue of Latin American underrepresentation in translational medicine and medical-clinical research, hindering the design of health policies targeted to their intended populations. This work demonstrates that bottom-up collaboration led to the creation of the Chilean COVID-19 Biorepository, providing a model for countries lacking governmental policies to support the installation and development of biobanks and biobank networks. However, the absence of such support not only hinders their creation but also threatens their long-term sustainability. While the C19-GenoNet network includes a sustainability strategy in its governance, the full responsibility for these nationally significant initiatives cannot rest solely on institutions or individual researchers, regardless of their motivation.

The lack of state policies in this field in Latin America delays the progress of translational, an issue that requires urgent correction. This work reaffirms the urgency of governmental support and highlights the potential for researchers to drive collaborative efforts in establishing biobanks and biobank networks. These networks are essential for providing access to high-quality, standardized samples and data, thereby enhancing research outcomes in precision and personalized medicine.

## Figures and Tables

**Table 1:** Average (SD) of ancestry ^a^ based on self-declared ethnicity.

Self-Reported Ethnicity	*n*	African (AFR)	Asian (EAS)	European (EUR)	Northern Andes	Southern Chile	Amerindian ^d^
African	6	24.8 (32.8)	1.0 (1.8)	36.2 (16.7)	23.5 (14.1)	14.5 (8.7)	37.9 (17.3)
Native American	252	1.4 (1.8)	0.9 (1.4)	36.5 (18.5)	24.6 (32.5)	36.6 (24.1)	61.2 (19.5)
Andes North ^b^	79	1.9 (2.6)	0.9 (2.0)	22.9 (20.1)	64.4 (31.0)	10.0 (9.5)	74.4 (22.4)
Chile South ^c^	162	0.9 (0.9)	0.8 (1.0)	42.4 (14.2)	5.6 (6.1)	50.3 (17.7)	55.9 (14.8)
Diaguita	11	4.2 (1.3)	1.1 (1.0)	48.8 (6.5)	19.5 (4.2)	26.5 (5.3)	45.9 (6.0)
Rapa Nui	2	7.7 (8.4)	1.0 (1.1)	41.3 (29.9)	28.5 (28.3)	21.5 (7.9)	50.0 (20.4)
Other	3	2.3 (0.6)	1.7 (2.1)	44.3 (22.7)	23.8 (28.5)	28.0 (10.4)	51.7 (20.9)
None Declared	1836	2.0 (3.1)	1.7 (4.5)	54.7 (13.2)	11.9 (13.2)	29.8 (13.1)	41.7 (13.2)
Whole Cohort	2099	2.0 (3.6)	1.6 (4.2)	52.4 (15.2)	13.4 (17.2)	30.6 (15.0)	44.0 (15.5)

^a^ Ancestry was estimated using ADMIXTURE on genotypes generated with the GSA v3 microarray; ^b^ Self-identified as Atacameño, Aymara, Quechua, or Colla; ^c^ Self-identified as Huilliche, Mapuche, or Kawésqar; ^d^ Amerindian ancestry for each participant was estimated by adding the Northern Andes and Southern Chile ancestries.

**Table 2:** Prevalence of the five most common symptoms and comorbidities reported in the survey and CRF for the whole biorepository and by severity level.

**Symptoms**					
Whole Biorepository		Mild		Hospitalized	
Fatigue	1541 (68.1%)	Fatigue	1355 (79.1%)	Dyspnea on exertion ^a^	98 (67.1%)
Headache	1521 (67.2%)	Headache	1326 (77.5%)	Pneumonia	88 (60.3%)
Myalgia	1365 (60.3%)	Anosmia	1193 (69.7%)	Fatigue	84 (57.5%)
Decay	1322 (58.4%)	Decay	1160 (67.8%)	Headache	82 (56.2%)
Anosmia	1305 (57.7%)	Myalgia	1157 (67.6%)	Fever	80 (54.8%)
Severe Hospitalized		Critically Ill		Lethal	
Dyspnea on exertion ^a^	58 (81.7%)	Dyspnea on exertion ^a^	107 (70.9%)	Dyspnea on exertion ^a^	7 (53.8%)
Fatigue	57 (80.3%)	Myalgia	83 (55.0%)	Fever	5 (38.5%)
Pneumonia	56 (78.9%)	Fever	81 (53.6%)	Persistent dry cough	3 (23.1%)
Difficulty breathing at rest	51 (71.8%)	Other symptoms	81 (53.6%)	New productive cough	2 (15.4%)
Decay	51 (71.8%)	Headache	65 (43.0%)	Diarrhea	2 (15.4%)
**Comorbidities**					
Whole Biorepository		Asymptomatic		Mild	
Other chronic diseases	719 (31.8%)	Obesity	55 (32.5%)	Other chronic diseases	571 (33.4%)
Obesity	691 (30.5%)	Other chronic diseases	38 (22.5%)	Obesity	529 (30.9%)
Hypertension	394 (17.4%)	Hypertension	27 (16.0%)	Hypertension	212 (12.4%)
Mental health problems	233 (10.3%)	Diabetes	21 (12.4%)	Mental health problems	185 (10.8%)
High cholesterol	232 (10.3%)	Mental health problems	17 (10.1%)	High cholesterol	161 (9.4%)
Hospitalized		Severe Hospitalized		Critically Ill	
Other chronic diseases	51 (34.9%)	Obesity	32 (45.1%)	Hypertension	72 (47.7%)
Hypertension	49 (33.6%)	Hypertension	26 (36.6%)	Diabetes	35 (23.2%)
Obesity	39 (26.7%)	Other chronic diseases	24 (33.8%)	Obesity	35 (23.2%)
Diabetes	28 (19.2%)	Diabetes	23 (32.4%)	Other chronic diseases	31 (20.5%)
High cholesterol	25 (17.1%)	High cholesterol	12 (16.9%)	High cholesterol	17 (11.3%)
Lethal					
Hypertension	8 (61.5%)				
Other chronic diseases	4 (30.8%)				
Cardiac problems	3 (23.1%)				
Diabetes	2 (15.4%)				
Coronary atherosclerosis	2 (15.4%)				

^a^ Sensation of running out of air during physical activity like walking up a flight of stairs.

## Data Availability

Researchers from any location can submit applications to access the Chilean COVID-19 Biorepository. Data and samples may be made available upon request and after ethical and scientific evaluation and approval to ensure compliance with national laws and relevant policies of the biobanks, institutions, and researchers involved in creating the biorepository and members of the C19-GenoNet biobank network. Individual-level ancestry estimates for European, African, Asian, and Amerindian origins are available along with demographic and clinically relevant variables.

## Supplementary Materials

The following supporting information can be downloaded at: https://www.mdpi.com/article/10.3390/genes15111352/s1, Table S1: Prevalence of symptoms reported by donors in the whole biorepository and by severity level; Table S2: Prevalence of reported comorbidities in the whole biorepository and by severity level.
