# Supplementary material for: The Chilean COVID-19 Genomics Network Biorepository: A Resource for Multi-Omics Studies of COVID-19 and Long COVID in a Latin American Population
Source: Genes (Basel). 2024 Oct 22;15(11):1352. doi: 10.3390/genes15111352 (PMC11593408; doi:10.3390/genes15111352)
Supplement: Supplementary file 1 [file genes-15-01352-s001.zip › genes-3018048-supplementary.pdf]

Table S1.
Prevalence of symptoms reported by donors in the whole biorepository and by severity level.

	Biorepository	Mild	Hospitalized	Severe hospitalized	Critically ill	Lethal
Fatigue	1541 (68.1%)	1355 (79.1%)	84 (57.5%)	57 (80.3%)	44 (29.1%)	1 (7.7%)
Headache	1521 (67.2%)	1326 (77.5%)	82 (56.2%)	47 (66.2%)	65 (43.0%)	1 (7.7%)
Myalgia	1365 (60.3%)	1157 (67.6%)	75 (51.4%)	48 (67.6%)	83 (55.0%)	2 (15.4%)
Decay	1322 (58.4%)	1160 (67.8%)	73 (50.0%)	51 (71.8%)	37 (24.5%)	1 (7.7%)
Anosmia	1305 (57.7%)	1193 (69.7%)	53 (36.3%)	29 (40.8%)	29 (19.2%)	1 (7.7%)
Ageusia	1165 (51.5%)	1064 (62.1%)	49 (33.6%)	28 (39.4%)	23 (15.2%)	1 (7.7%)
Dyspnea on exertion[a]	1054 (46.6%)	784 (45.8%)	98 (67.1%)	58 (81.7%)	107 (70.9%)	7 (53.8%)
Persistent dry cough	988 (43.7%)	806 (47.1%)	78 (53.4%)	38 (53.5%)	63 (41.7%)	3 (23.1%)
Fever	968 (42.8%)	754 (44.0%)	80 (54.8%)	48 (67.6%)	81 (53.6%)	5 (38.5%)
Chills	951 (42.0%)	823 (48.1%)	62 (42.5%)	41 (57.7%)	24 (15.9%)	1 (7.7%)
Sore throat	844 (37.3%)	752 (43.9%)	36 (24.7%)	32 (45.1%)	24 (15.9%)	0 (0.0%)
Heat and cold sensation	806 (35.6%)	708 (41.4%)	40 (27.4%)	37 (52.1%)	21 (13.9%)	0 (0.0%)
Back pain	801 (35.4%)	693 (40.5%)	45 (30.8%)	32 (45.1%)	31 (20.5%)	0 (0.0%)
Nasal congestion	781 (34.5%)	720 (42.1%)	23 (15.8%)	18 (25.4%)	20 (13.2%)	0 (0.0%)
Diarrhea	742 (32.8%)	652 (38.1%)	39 (26.7%)	21 (29.6%)	28 (18.5%)	2 (15.4%)
Chest tightness	694 (30.7%)	568 (33.2%)	54 (37.0%)	39 (54.9%)	32 (21.2%)	1 (7.7%)
Difficulty breathing at rest	678 (30.0%)	501 (29.3%)	75 (51.4%)	51 (71.8%)	50 (33.1%)	1 (7.7%)
Red eyes	599 (26.5%)	533 (31.1%)	22 (15.1%)	25 (35.2%)	19 (12.6%)	0 (0.0%)
Other symptoms	589 (26.0%)	438 (25.6%)	49 (33.6%)	19 (26.8%)	81 (53.6%)	2 (15.4%)
Dizziness	522 (23.1%)	444 (25.9%)	34 (23.3%)	28 (39.4%)	16 (10.6%)	0 (0.0%)
Numbness	450 (19.9%)	363 (21.2%)	32 (21.9%)	32 (45.1%)	23 (15.2%)	0 (0.0%)
Abdominal pain	440 (19.5%)	371 (21.7%)	23 (15.8%)	25 (35.2%)	20 (13.2%)	1 (7.7%)
Vomiting	439 (19.4%)	379 (22.1%)	28 (19.2%)	19 (26.8%)	13 (8.6%)	0 (0.0%)
Feeling of heavy limbs	439 (19.4%)	369 (21.6%)	24 (16.4%)	27 (38.0%)	19 (12.6%)	0 (0.0%)
Sneezing	397 (17.6%)	359 (21.0%)	15 (10.3%)	15 (21.1%)	8 (5.3%)	0 (0.0%)
Painful breathing	386 (17.1%)	304 (17.8%)	35 (24.0%)	30 (42.3%)	17 (11.3%)	0 (0.0%)
Rhinorrhea	367 (16.2%)	324 (18.9%)	15 (10.3%)	16 (22.5%)	12 (7.9%)	0 (0.0%)
New productive cough	362 (16.0%)	294 (17.2%)	25 (17.1%)	15 (21.1%)	26 (17.2%)	2 (15.4%)
Inability to move	341 (15.1%)	256 (15.0%)	28 (19.2%)	34 (47.9%)	23 (15.2%)	0 (0.0%)
Pneumonia	283 (12.5%)	95 (5.5%)	88 (60.3%)	56 (78.9%)	43 (28.5%)	1 (7.7%)
Erythema	148 (6.5%)	124 (7.2%)	12 (8.2%)	8 (11.3%)	4 (2.6%)	0 (0.0%)
Eruptions in mouth	130 (5.7%)	114 (6.7%)	8 (5.5%)	4 (5.6%)	4 (2.6%)	0 (0.0%)
Skin Rashes	110 (4.9%)	86 (5.0%)	7 (4.8%)	12 (16.9%)	5 (3.3%)	0 (0.0%)
Skin discoloration	68 (3.0%)	47 (2.7%)	12 (8.2%)	2 (2.8%)	7 (4.6%)	0 (0.0%)

[a] Sensation of running out of air during physical activity like walking up a flight of stairs

Table S2. Prevalence of reported comorbidities in the whole biorepository and by severity level.

	**Biorepository**	**Asymptomatic**	**Mild**	**Hospitalized**	**Severe hospitalized**	**Critically ill**	**Lethal**
Other chronic diseases	719 (31.8%)	38 (22.5%)	571 (33.4%)	51 (34.9%)	24 (33.8%)	31 (20.5%)	4 (30.8%)
Obesity	691 (30.5%)	55 (32.5%)	529 (30.9%)	39 (26.7%)	32 (45.1%)	35 (23.2%)	1 (7.7%)
Hypertension	394 (17.4%)	27 (16%)	212 (12.4%)	49 (33.6%)	26 (36.6%)	72 (47.7%)	8 (61.5%)
Mental health problems	233 (10.3%)	17 (10.1%)	185 (10.8%)	14 (9.6%)	6 (8.5%)	11 (7.3%)	0 (0.0%)
High cholesterol	232 (10.3%)	17 (10.1%)	161 (9.4%)	25 (17.1%)	12 (16.9%)	17 (11.3%)	0 (0.0%)
Diabetes	212 (9.4%)	21 (12.4%)	103 (6.0%)	28 (19.2%)	23 (32.4%)	35 (23.2%)	2 (15.4%)
Asthma	148 (6.5%)	9 (5.3%)	108 (6.3%)	13 (8.9%)	6 (8.5%)	11 (7.3%)	1 (7.7%)
Cancer (several types)	98 (4.3%)	4 (2.4%)	69 (4.0%)	15 (10.3%)	2 (2.8%)	8 (5.3%)	0 (0.0%)
Anemia	87 (3.8%)	2 (1.2%)	72 (4.2%)	9 (6.2%)	2 (2.8%)	2 (1.3%)	0 (0.0%)
Cardiac problems	65 (2.9%)	5 (3.0%)	37 (2.2%)	9 (6.2%)	3 (4.2%)	8 (5.3%)	3 (23.1%)
Hepatitis	39 (1.7%)	1 (0.6%)	30 (1.8%)	4 (2.7%)	3 (4.2%)	0 (0.0%)	1 (7.7%)
Vascular accident	33 (1.5%)	3 (1.8%)	18 (1.1%)	6 (4.1%)	2 (2.8%)	4 (2.6%)	0 (0.0%)
Weakened immune system	27 (1.2%)	1 (0.6%)	21 (1.2%)	1 (0.7%)	1 (1.4%)	2 (1.3%)	1 (7.7%)
Rheumatoid arthritis	28 (1.2%)	3 (1.8%)	18 (1.1%)	3 (2.1%)	2 (2.8%)	2 (1.3%)	0 (0.0%)
Coronary atherosclerosis	25 (1.1%)	0 (0.0%)	16 (0.9%)	2 (1.4%)	1 (1.4%)	4 (2.6%)	2 (15.4%)
Pulmonary condition	25 (1.1%)	0 (0.0%)	17 (1.0%)	1 (0.7%)	1 (1.4%)	5 (3.3%)	1 (7.7%)
Other rheumatoid diseases	25 (1.1%)	1 (0.6%)	21 (1.2%)	2 (1.4%)	0 (0.0%)	1 (0.7%)	0 (0.0%)
Dialysis	13 (0.6%)	0 (0.0%)	6 (0.4%)	3 (2.1%)	0 (0.0%)	2 (1.3%)	2 (15.4%)
Brain pathology	12 (0.5%)	3 (1.8%)	4 (0.2%)	1 (0.7%)	1 (1.4%)	3 (2.0%)	0 (0.0%)
HIV	12 (0.5%)	0 (0.0%)	7 (0.4%)	3 (2.1%)	0 (0.0%)	1 (0.7%)	1 (7.7%)
Ulcerative colitis or Crohn's disease	12 (0.5%)	0 (0.0%)	9 (0.5%)	2 (1.4%)	1 (1.4%)	0 (0.0%)	0 (0.0%)
Lupus	7 (0.3%)	0 (0.0%)	6 (0.4%)	0 (0.0%)	1 (1.4%)	0 (0.0%)	0 (0.0%)
Pulmonary fibrosis	5 (0.2%)	0 (0.0%)	2 (0.1%)	1 (0.7%)	1 (1.4%)	1 (0.7%)	0 (0.0%)
Tuberculosis	5 (0.2%)	2 (1.2%)	3 (0.2%)	0 (0.0%)	0 (0.0%)	0 (0.0%)	0 (0.0%)
Cystic fibrosis	2 (0.1%)	0 (0.0%)	2 (0.1%)	0 (0.0%)	0 (0.0%)	0 (0.0%)	0 (0.0%)
Transplant of an organ	2 (0.1%)	0 (0.0%)	2 (0.1%)	0 (0.0%)	0 (0.0%)	0 (0.0%)	0 (0.0%)
